# Asking the right questions: towards a person-centered conception of shared decision-making regarding treatment of advanced chronic kidney disease in older patients

**DOI:** 10.1186/s12910-022-00784-x

**Published:** 2022-04-27

**Authors:** Wouter R. Verberne, Anne M. Stiggelbout, Willem Jan W. Bos, Johannes J. M. van Delden

**Affiliations:** 1 Department of Internal Medicine, St Antonius Hospital, Nieuwegein, The Netherlands; 2 Department of Internal Medicine, Leiden University Medical Center, Leiden, The Netherlands; 3 Department of Internal Medicine, University Medical Center Utrecht, Heidelberglaan 100, 3584 CX Utrecht, The Netherlands; 4 Medical Decision Making, Department of Biomedical Data Sciences, Leiden University Medical Center, Leiden, The Netherlands; 5 University Medical Center Utrecht, Julius Center for Health Sciences and Primary Care, Utrecht, The Netherlands

**Keywords:** Shared decision-making, Older patients, Dialysis, Conservative care, Person-centered care

## Abstract

An increasing number of older patients have to decide on a treatment plan for advanced chronic kidney disease (CKD), involving dialysis or conservative care. Shared decision-making (SDM) is recommended as the model for decision-making in such preference-sensitive decisions. The aim of SDM is to come to decisions that are consistent with the patient’s values and preferences and made by the patient and healthcare professional working together. In clinical practice, however, SDM appears to be not yet routine and needs further implementation. A shift from a biomedical to a person-centered conception might help to make the process more shared. Shared should, therefore, be interpreted as two persons bringing two perspectives to the table, that both need to be explored during the decision-making process. Starting from the patient’s perspective will enable to determine the mutual goals of care first and, subsequently, determine the best way for achieving those goals. To perform such SDM, the healthcare professional needs to become a skilled companion, being part of the patient’s relational context, and start asking the right questions about what matters to the patient as person. In this article, we describe the need for a person-centered conception of SDM for the setting of older patients with advanced CKD.

## Background

### Older patients with advanced chronic kidney disease

The number of older patients with advanced chronic kidney disease (CKD) has increased considerably in recent years [1, 2]. Among older patients, dialysis has become an established practice and is nowadays the most common treatment pathway in this setting in many countries [3–5]. Some older patients, however, do consider dialysis as too burdensome, questioning whether the potential benefits of dialysis outweigh its intensive therapy regimen [6–9]. Based on emerging evidence, conservative care has been recognized as a viable treatment alternative to dialysis in selected older patients [10–12]. Particularly in patients with the highest ages or multiple comorbid conditions, a choice for conservative care instead of dialysis has potential to achieve similar survival and health-related quality of life outcomes at lower treatment burden [13–15]. Conservative care involves all active medical treatment and multidisciplinary care except for dialysis, and is intended to be provided until death [10]. While the decision to forego dialysis was already a legitimate and justifiable option before [16–18], the more widespread recognition of conservative care now offers more older patients and healthcare professionals a valuable alternative to dialysis [10–12].

Shared decision-making (SDM) is recommended as the model for decision-making in preference-sensitive decisions [19, 20]. Older patients with advanced CKD have to make such a decision. Although an uniformly accepted definition of SDM is lacking [21], the main goal of SDM is to enable decision-making by the patient and healthcare professional working together on a treatment or care plan that is consistent with the patient’s values and preferences and also professionally acceptable. Its fundamental idea is that the patient is the expert on what matters in their life and the healthcare professional is the expert on biomedical evidence. Hence, SDM aims to combine the unique expertise of both patient and healthcare professional.

### Current decision-making

Recently, we examined experiences of older patients with a decision-making practice regarding dialysis or conservative care in which we strived to achieve a process of SDM [7]. Patients indicated to be overall satisfied with the decision-making process and their treatment decision. However, patients reported negative decision-making experiences as well, especially patients who had chosen dialysis. A major finding was that patients felt unprepared, overwhelmed, or even forced to decide, despite the decision-making process having been started early by an experienced multidisciplinary team and having involved various interactions. Apparently, these patients had still been insufficiently aware of their situation and disease course. Some patients also described a mismatch between their preference for (often more) and perceived participation (often too limited) in the decision-making about treatment plan, including a perceived lack of own choice.

The findings of our single-center study do not stand on their own. More studies found patients describing poor decision-making experiences, and also particularly in patients who chose the most intensive treatment pathways, including dialysis [8, 22–25]. In current practice, patients often indicate to experience a lack of power, despite being informed about possible treatment options [26, 27]. Patients also often describe that they feel unprepared, not confident, or incompetent to participate in the decision-making about their treatment or care plan [22, 23, 26, 28]. At the same time, studies observe that a substantial part of patients still doubts or even regrets their treatment decision afterwards, especially if the decision was more driven by the healthcare professional’s preference [29–31]. Many patients express the need for more time and particularly more interactions with their healthcare team to consider more thoroughly their situation, their options, and what matters to them, as well as to be more fully seen and heard [28, 32–35].

### Towards improved decision-making

Despite efforts to implement SDM and improve patient involvement, SDM appears to be not yet routine or executed adequately in current clinical practice [19, 26, 36–38]. Multiple barriers and facilitators have been identified to foster further implementation, varying from system-level and organizational factors to interaction factors during the decision-making process, such as pre-disposing patient characteristics and the healthcare professional’s approach towards SDM and patient involvement [26, 39–42]. Building on recent findings [43, 44], we think that another important barrier in current decision-making practices might be too strong a focus on the biomedical perspective and SDM as technique, resulting in “shared” decision-making between treatment options that is predominantly driven by biomedical reasoning and with too limited attention for effective partnership building between the patient and healthcare professional. A shift from a biomedical and technical orientation to a person-centered orientation of SDM might improve current decision-making practices, and has recently been proposed for SDM in patients with multiple comorbid conditions and other complex chronic care situations [45–48]. The aim of this article is, therefore, to reconsider how decision-making regarding treatment in older patients with advanced CKD could become more person-centered and, overall, more truly shared.

## Main body

### Reconsidering shared decision-making

#### The shared decision-making model

SDM is often perceived as the middle between the paternalistic and informed models of decision-making [49]. In the paternalistic model, the healthcare professional decides what is best for the patient, based on their judgment about beneficence and non-maleficence. Major criticism of this model is its failure to respect the patient’s autonomy and the patient’s moral right to self-determination. In the informed model, the patient independently decides after being informed by the healthcare professional. Major criticisms of this model are the limited role of the healthcare professional, restricted to informing only, and its high demands on patients, who often find themselves struggling with making their own decision [22, 23, 26, 50]. The SDM model recognizes the need for involvement of both the patient and the healthcare professional, and stresses their partnership in decision-making [21, 51, 52]. Such partnership involves a dialogue about what matters to the patient, and of pros and cons of possible options. Hence, SDM is about sharing different perspectives and sharing the process of decision-making, with the aim to respect and also foster the patient’s autonomy [51, 53].

#### Perspectives in shared decision-making

Since the healthcare professional is part of the relational context of the patient, building an effective partnership is essential for SDM as well as the involvement of both their unique perspectives [49]. Traditionally, SDM starts from the healthcare professional’s biomedical perspective, in which the possible medical options for a given disease or condition are being considered first (*e.g.*, the option talk in several SDM models [19, 21, 54]). The perspective from which SDM starts, however, matters since it may bring a specific orientation or frame in which SDM further takes place. Starting SDM from a biomedical perspective directly brings a focus on which medical options are effective for treating an, often single, condition (*e.g.*, disease X or symptom Y). In such orientation, the patient and healthcare professional are most likely to discuss and deliberate on the patient’s perspective *after* the healthcare professional has brought to the table their expertise on the possible medical options first [19]. Such decision-making process is reasonable in the context of acute diseases. In older patients with advanced CKD, starting SDM from a biomedical perspective, however, will bring a focus on the available treatment options, including dialysis and conservative care, and emphasizes the need for the healthcare professional to inform the patient about these options including their pros and cons. While informing the patient is important, a potential pitfall of this approach is too strong a focus on the biomedical perspective and hence, treatment. Thereby, the decision-making process could become rather dominated by the healthcare professional’s contribution and limit the involvement of the patient’s unique perspective. Furthermore, clearly informing the patient about possible treatment options itself is difficult and often not as neutral or unbiased as might be assumed [55, 56]. Healthcare professionals unwittingly frame the information and thus the decision-making in their attempt to inform the patient, which could undermine the patient’s autonomy, for example by what information about possible care options is exactly presented, in what order, and in what way—both verbally and non-verbally [57].

Starting SDM from the patient’s perspective has recently been proposed for SDM in patients with multiple comorbid conditions and other complex chronic care situations, which often involve contexts of full uncertainty [46–48]. This SDM approach is based on a person-centered orientation [45, 58, 59]. Person-centered care describes the importance of knowing the person behind the patient, in order to empower the person to actively take part in finding ways to achieve the goals that matter to that person [60–64]. A person-centered approach of SDM, therefore, needs the patient and healthcare professional to gain understanding of what matters to the patient first. Then, by combining the patient’s expertise based on their lived experience with the healthcare professional’s expertise, the patient and healthcare professional become able to determine what goals of care are important and, subsequently, what options could help best to achieve those goals. Hence, a person-centered orientation of SDM involves a shift from figuring out “What is the matter with you?” and the aim to fix that, to “What matters to you?” in order to determine the best way to act [64, 65].

While person-centered care and SDM are often described as closely linked to each other [59, 65], these concepts are in fact not the same and do not necessarily co-exist [43, 44]. Instead, what currently is interpreted as high-quality SDM does not need person-centered elements to be involved [43]. Such elements include respect for the patient as person, compassion and empathy and are needed to build effective partnerships between the patient and the healthcare professional and help create the right conditions for active patient involvement [44, 63, 64, 66]. The main focus in SDM literature and implementation, however, is on technique, including the correct sequence of steps required to perform high-quality SDM, rather than person-centeredness [43, 59, 67]. As a result, we think that the focus on technique also emphasizes the need in clinical practice to start SDM from the healthcare professional’s biomedical perspective.

Starting SDM from the patient’s perspective is, however, particularly needed in complex chronic care contexts, in order to help decide what options could contribute to the patient’s life in a meaningful way. In these contexts, starting from a focus on the person could help bring all relevant information to the table including the patient’s perspective and build a more equal and effective partnership between patient and healthcare professional. Such orientation and connection are essential to learn about the specific goals of care that matter to the patient given their situation (*e.g.*, an older and frail patient with multiple comorbid conditions) before potential care or treatment options are considered. Hence, a person-centered approach requires a different process of SDM, in which deciding on treatment is preceded by development of mutual goals of care first, and for which both the patient’s and healthcare professional’s expertise are needed. Especially in complex chronic care contexts, the patient and healthcare professional thereby become able to consider what way is best to enable that person to do the things in life that matter to them, and, overall, how to be resilient and adaptive while having multiple chronic conditions [68–72].

#### The context of older patients with advanced chronic kidney disease

In older patients with advanced CKD, starting SDM from the patient’s perspective is also more likely to enable an open-ended dialogue. Such person-centered orientation could help the patient to better understand their situation first, the healthcare professional to learn about the person behind the patient, and overall help build an effective partnership based on safety, respect and trust required for active patient involvement [73]. Such approach in this complex chronic care context is particularly relevant to align care more with personal priorities, since many older patients with advanced CKD have multiple comorbid conditions, functional impairments, are frail and approaching end of life [74]. In our study on decision-making in older patients with advanced CKD, we observed that patients had diverse and contrasting reasons for their decision [7]. Moreover, most patients considered their personal values and preferences regarding life, quality of life, and death more important than biomedical factors such as treatment effectiveness. Other studies showed that nephrologists still predominantly base their treatment recommendation on biomedical factors and a tendency to prolong life [23, 39, 40], even if they prioritize the patient’s values and preferences as most important [75, 76]. Furthermore, nephrologists appear to have limited knowledge of what patients consider to be important [77].

Therefore, a shift is needed from a biomedical and technical conception of SDM to a person-centered conception in SDM practices with older patients with advanced CKD, which aims to better combine the unique expertise of both the patient and healthcare professional and establish a more effective and equal partnership. Shared does not imply two persons considering the same perspective but rather two perspectives that both need to be explored and developed during the decision-making process. In this chronic care setting, SDM should start from the patient’s perspective, in order to become person-centered and, overall, to come to a treatment plan that fits best with the goals that matter to that specific person.

#### Towards a person-centered conception of shared decision-making

An important question now is what a person-centered conception of SDM means for clinical practice, and specifically for the context of older patients with advanced CKD. New SDM models that describe a person-centered and goal-based approach have recently been developed for the contexts of patients with multiple comorbid conditions and other complex chronic care situations [47, 48]. For example, the three-talk-model of SDM by Elwyn et al. has been integrated with a model for upfront goal setting, which starts from the patient’s perspective [47]. But what elements are needed to enable such person-centered approaches of SDM in clinical practice? We describe three main elements needed: (1) asking the right questions, (2) a dynamic decision-making process, and (3) skilled companionship by the healthcare professionals involved.

1. Asking the right questions

To shift to a person-centered orientation of SDM, healthcare professionals such as nephrologists and the multidisciplinary team of nurses and social workers should focus more on asking the right questions, next to providing biomedical information. These questions serve to learn about the person behind the patient, help build an effective partnership, and help develop the patient’s perspective on all that matters. A person-centered approach aims to develop answers to questions like: Who are you? What matters to you? What does your situation, which might involve multiple chronic conditions and an approaching end of life, mean to you? And what do you want to be able to achieve to in life given the fact of your situation? Hence, person-centered SDM requires a reflection discourse, rather than an information and decision discourse [78]. Such an approach should allow languages other than medical language only, in order to include all relevant narratives [27, 64, 79]. While the use of patient-reported outcome measures may help to identify less routinely discussed topics that matter to a patient, most still have a biomedical orientation. Additional approaches are needed to ask all the right questions that focus on the person. A new concept of health such as that of positive health could help healthcare professionals in this process [72, 80]. Given the rise of chronic disease, positive health offers a more contemporary and dynamic definition of health as the ability to adapt and to self-manage, opposed to the static WHO definition of health as complete wellbeing [72]. The concept of positive health has been developed by input of multiple stakeholders, including patients, and involves the aim to start a dialogue about what goals of care matter by focusing on what the patient is still able to do and wants to achieve [81]. Particularly in older and frail patients, such development of goals of care based on positive health might help to determine the best way to support the older patient’s resilience and ability to adapt given their situation.

Although more challenging, developing an understanding of the patient’s perspective remains essential for healthcare professionals to strive to in patients who do not prefer or are unable to decide themselves, in order to determine the best way to act consistently with what matters to the patient. Also more challenging might be if the patient and healthcare professional hold different values or disagree about what goals of care matter. Such differences, however, offer a valuable starting point for further discussion and development of both perspectives, which is in fact essential to SDM.

(2) Dynamic decision-making process

To further enable a person-centered approach of SDM, a dynamic and ongoing process is needed in which the patient and healthcare professional have sufficient time and opportunities for interaction and building an effective partnership [7]. As in advance care planning, such a process of decision-making should be started before a decision on treatment is needed, in order to enable a more open-ended dialogue on the patient’s situation and life first. The chronic and often slowly progressive disease course in advanced CKD offers valuable opportunities to start such dynamic process of SDM. A timely started process also enables more ongoing development and evaluation of what matters to the patient, since this may change over time, particularly after changes in health or personal situation [9, 73, 82]. Subsequently, when a decisional moment on treatment plan approaches, decision-making could become more targeted on what treatment plan would be best to achieve the goals that matter to the patient.

(3) Skilled companionship

In person-centered SDM, an effective and equal partnership between the patient and healthcare professional is needed that aims to foster the patient’s autonomy [49, 64]. Therefore, the healthcare professional should become a skilled companion to the patient to help create the right conditions for autonomous choices [83]. Skilled in supporting the patient during the entire disease course and decision-making process with person-centered communication elements, including asking the right questions, actively helping to develop the patient’s perspective on what matters, and in adequately sharing their biomedical expertise. Companionship as in walking together with the patient during the disease trajectory, including timely addressing potential changes in the disease course and prognosis, also if this involves end of life issues, in order to enable a more ongoing dialogue about the patient’s situation [39, 40, 84, 85]. The long-term relationship between older patients and their multidisciplinary healthcare team in advanced CKD care offers valuable opportunities to establish partnerships based on such skilled companionship.

### Further development

Research and clinical efforts are needed to further develop person-centered approaches of SDM for chronic care settings, such as that of older patients with advanced CKD. All relevant stakeholders, including patients, should be involved in such efforts. One of the main tasks is to develop interventions and resources for the healthcare professional to enable their more active role as skilled companion in the SDM process. Several tools are already available that could help to ask the right questions and develop a shared understanding of what matters to the patient [47, 86], including tools to reflect on the dimensions of positive health and to help prioritize goals of care [77, 81, 87, 88]. Research and evaluations are also needed to determine what interventions are adequate and reasonable for fostering the patient’s autonomy [49]. Since autonomy is dependent of the relational context [51], the forms of support needed differ per situation, patient, and healthcare professional involved, and should therefore be applied and evaluated accordingly in a contextually sensitive way [52]. Also, the roles of the patient’s family and other contextual partners need further elaboration [50]. Furthermore, training and education for healthcare professionals is needed to improve understanding what SDM and person-centered care are, why SDM and person-centered care are needed, and how person-centered SDM should be performed in complex chronic care settings [64, 89].

## Conclusions

SDM is a valuable model for decision-making to help decide on a treatment plan that is consistent with what matters to the patient and also professionally acceptable. In older patients with advanced CKD, a shift in clinical practice is needed, although, from a biomedical and technical conception of SDM to a person-centered conception, which aims to better combine the unique expertise of both the patient and healthcare professional and help build a more equal and effective partnership. Shared should, therefore, be interpreted as two persons bringing two perspectives to the table, that both need to be explored during the decision-making process. Furthermore, starting SDM from the patient’s perspective, rather than from the healthcare professional’s biomedical perspective, is likely to enable a more open-ended dialogue about all that matters to the patient as person. Thereby, the patient and healthcare professional become able to determine the goals of care, followed by what options could help best for achieving those goals. Hence, a person-centered approach requires a dynamic process of SDM in which deciding on treatment is preceded by development of mutual goals of care first. In this chronic care setting, a person-centered approach is particularly relevant to help consider the best way to enable the older and often frail patient to do the things in life that matter to them, and for supporting the patient’s resilience given their situation. To enable such goal-based SDM, the healthcare professional needs to become a skilled companion to the patient, being part of the patient’s relational context, and start asking the right questions about what matters to the patient.

## Data Availability

Not applicable.

## Abbreviations

CKD: Chronic kidney disease
SDM: Shared decision-making
